# Inferring the Ecological Niche of *Toxoplasma gondii* and *Bartonella* spp. in Wild Felids

**DOI:** 10.3389/fvets.2017.00172

**Published:** 2017-10-17

**Authors:** Luis E. Escobar, Scott Carver, Daniel Romero-Alvarez, Sue VandeWoude, Kevin R. Crooks, Michael R. Lappin, Meggan E. Craft

**Affiliations:** ^1^Department of Veterinary Population Medicine, University of Minnesota, Minneapolis, MN, United States; ^2^Department of Fisheries, Wildlife and Conservation Biology, University of Minnesota, St. Paul, MN, United States; ^3^Department of Fish and Wildlife Conservation, Virginia Tech, Blacksburg, VA, United States; ^4^School of Biological Sciences, University of Tasmania, Hobart, TAS, Australia; ^5^Department of Ecology and Evolutionary Biology, University of Kansas, Lawrence, KS, United States; ^6^Department of Microbiology, Immunology and Pathology, Colorado State University, Fort Collins, CO, United States; ^7^Department of Fish, Wildlife, and Conservation Biology, Colorado State University, Fort Collins, CO, United States; ^8^Department of Clinical Sciences, Colorado State University, Fort Collins, CO, United States

**Keywords:** *Bartonella* spp., environmental transmission, *Lynx rufus*, niche, *Puma concolor*, *Toxoplasma gondii*

## Abstract

Traditional epidemiological studies of disease in animal populations often focus on directly transmitted pathogens. One reason pathogens with complex lifecycles are understudied could be due to challenges associated with detection in vectors and the environment. Ecological niche modeling (ENM) is a methodological approach that overcomes some of the detection challenges often seen with vector or environmentally dependent pathogens. We test this approach using a unique dataset of two pathogens in wild felids across North America: *Toxoplasma gondii* and *Bartonella* spp. in bobcats (*Lynx rufus*) and puma (*Puma concolor*). We found three main patterns. First, *T. gondii* showed a broader use of environmental conditions than did *Bartonella* spp. Also, ecological niche models, and Normalized Difference Vegetation Index satellite imagery, were useful even when applied to wide-ranging hosts. Finally, ENM results from one region could be applied to other regions, thus transferring information across different landscapes. With this research, we detail the uncertainty of epidemiological risk models across novel environments, thereby advancing tools available for epidemiological decision-making. We propose that ENM could be a valuable tool for enabling understanding of transmission risk, contributing to more focused prevention and control options for infectious diseases.

## Introduction

Traditional epidemiological studies of disease in animal populations are dominated by intraspecific transmission of contact-dependent (directly transmitted) parasites or pathogens (1, 2). However, many important parasites have complex life cycles that include vectors or environmental stages, and we often know much less about these types of parasites (3). For parasites or pathogens transmitted *via* vectors or the environment, it is especially important to understand not only the relationships between the host and pathogen, but also the environmental niche—the environmental conditions in which the pathogen persists in the long term (4). In practice, understanding the environmental niche of many pathogens can be difficult to achieve due to challenges associated with detecting pathogens in vectors and the environment (e.g., sparsely distributed pathogens in vectors, in soil, on plant matter, or in water). As an alternative, capturing and sampling wildlife hosts is more effective. Innovative methodological approaches that overcome some of these environmental challenges are therefore needed and would be valuable for enabling understanding of transmission risk, thereby contributing to more focused prevention and control options.

Current approaches to map pathogens often include conducting a cluster analysis and spatial interpolations of disease cases in a specific area, thereby creating a tentative risk map for pathogen exposure (5, 6). However, a limitation of these classic approaches is the questionable value for forecasting risk in novel areas beyond those with ongoing surveillance. That is to say, geographic interpolations and cluster analyses do not consider environmental features and only reflect the sampling effort (7). Environmental (or ecological) niche modeling (ENM) is the practice of reconstructing a species’ environmental determinants (8). These methods can be useful in creating predictive maps that can forecast pathogen presence in novel regions (9). Ecological niche modeling is established for species distribution modeling and is gaining attraction in the field of veterinary epidemiology (7).

Recent research has demonstrated the utility of ENM to predict disease in distant novel areas (8), but it remains rare for these predictive models to be validated using independent data, which is particularly true for models of pathogens in wildlife. We tackle this problem using a unique dataset of two pathogens, *Toxoplasma gondii* and *Bartonella* spp., isolated from wild felids across North America. Specifically, we analyzed samples from bobcats (*Lynx rufus*) and puma (*Puma concolor*), two secretive carnivores that are widespread in North America and are adaptable to a wide array of habitats where they are exposed to pathogens acquired from their environment (10–12). *T. gondii* is an intracellular protozoan parasite found in warm-blooded animals, including birds and mammals, and is transmitted *via* consumption of sporulated oocysts in feces, water, and soil or bradyzoites in tissues of prey species (13); in these wild felids, *T. gondii* is likely transmitted *via* consumption of infected prey such as rodents, lagomorphs, and cervids (10). The *Bartonella* genus includes gram negative anaerobic facultative intracellular bacteria species that cause an array of diseases affecting mammals; contact with arthropod vectors, particularly fleas, is the primary route of transmission of *Bartonella henselae, Bartonella koehlerae*, and *Bartonella clarridgeiae* (hereafter *Bartonella* spp.) (14, 15). Both, *T. gondii* and *Bartonella* spp., require other organisms to persist; thus, here we define them as micro-parasites or simply parasites (7).

This study has two primary tasks. First, we evaluate if ENM can characterize the potential distribution of parasites with complex lifecycles found in felid host species. This is particularly important when there is limited knowledge about the environmental niche of the pathogens, such as in this study. Second, we examine if ENM results from one region can be applied to other novel regions. We emphasize important novelties from this study: (i) this study utilizes remote sensing data that captures the habitat heterogeneity across study sites with high detail; (ii) this environmental heterogeneity is explicitly incorporated into risk maps produced by ENM; and (iii) we detail the uncertainty of epidemiological risk models across novel environments, thereby advancing epidemiological decision-making tools.

## Materials and Methods

Our dataset included 467 felids serologically positive for *T. gondii* and/or *Bartonella* spp. from Florida, Colorado, and California. Of these exposed felids, 328 were positive to *T. gondii* parasites and 234 to *Bartonella* spp.; occurrence records contained each animal’s capture location and exposure status (16). These data were coupled with landscape information from satellite imagery to develop ENMs and create a risk map for each pathogen.

### Occurrences

Occurrences of *T. gondii* and *Bartonella* spp. were recorded in ongoing research featuring an unusually large collection of wild felid serosurvey data from three different study areas: Florida, Colorado, and California (10, 12, 17, 18). The study areas were chosen as part of a previous study to represent a range of sites important for puma and bobcat conservation and were also representative of a wide degree of anthropogenic impacts (i.e., habitat fragmentation, urbanization, and agriculture) across North America. The Californian study region is a highly urbanized landscape characterized by a warm dry Mediterranean climate with vegetation communities dominated by coastal California sage scrub, chaparral, riparian and coastal oak woodlands, and annual grasslands. Colorado region was delimited by two polygons resembling sampling in rural and exurban areas with cold semi-arid climates and vegetation characterized by coniferous woodlands and forests primarily interspersed with aspens. The two regions in Colorado represent an area proximate to human development and a more natural area with agricultural surroundings. The Florida region is a mixture of urban, exurban, and agricultural areas spanning humid subtropical and tropical savanna climates with vegetation communities consisting of pine flatwoods, south Florida rockland, cypress domes and strands, dwarf cypress, prairies, mixed hardwood swamps, hardwood hammocks, freshwater swamps, and mangroves.

At each region, individual felids were captured, their location recorded, and samples for pathogen screening were collected according to protocols previously described (10, 12, 18). Wild felids were anesthetized using various tranquilizers/sedatives (19, 20), sampled, and released. Thoracic fluid was collected from hunter-killed animals instead of serum for a subset of bobcats from Colorado (11). Blood and serum samples were initially stored in ethylenediaminetetraacetic acid and serum-separating tubes. Samples were either refrigerated at 4°C or kept on ice until return from the field where they were temporarily frozen at −20°C, and later transferred to −80°C until screening for pathogen exposure. All procedures were performed after appropriate Institutional Animal Care and Use Committee approvals were obtained.

Exposure to *T. gondii* and *Bartonella* spp. in puma (*P. concolor*) and bobcats (*L. rufus*) was estimated by measuring serum antibodies at the Specialized Infectious Disease Laboratory (Colorado State University) according to protocols previously described (10, 12). Serological samples were considered positive for *T. gondii* if they were positive to IgM or IgG. Samples were considered positive to *Bartonella* spp. if immunofluorescence antibody assay (IFA) tests detecting antibodies against *B. henselae* and *B. clarridgeiae* were positive (21–23); this was also confirmed independently by performing PCR on matched blood samples (12). For each study area and species, samples were generally collected over a 2- to 3-year intensive study period, and cumulatively the majority of samples across all sites were collected between 2001 and 2012 (12, 16). Puma and bobcat from Florida were not tested for *Bartonella* spp. (12). For the purpose of reducing overfit of models to the data, duplicate pathogen records from the same location (i.e., those from different individuals captured at the same location, but both exposed to the same pathogen) were restricted to single occurrence records for analyses.

### Model Calibration Area

**Figure 1:**
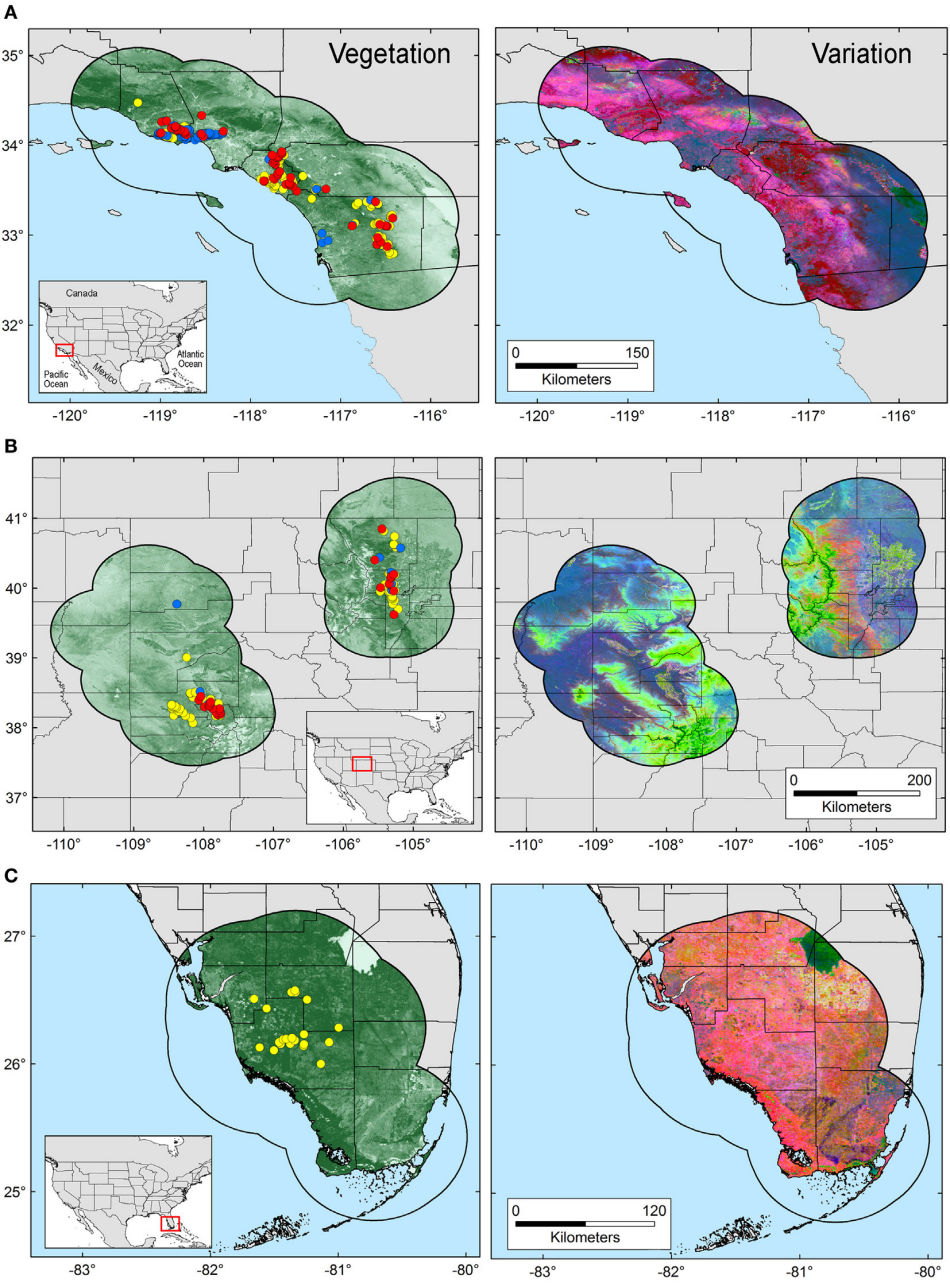
Study areas and environmental variables employed in this study. Model calibration areas were defined in California **(A)**, Colorado **(B)**, and Florida **(C)** based on a buffer zone estimated from the average distance among occurrences. Left: occurrences for *Toxoplasma gondii* (yellow points), *Bartonella* spp. (blue points), and co-infections (red points) are displayed on a surface resembling landscape vegetation in the form of Normalized Difference Vegetation Index (NDVI) data. Right: original NDVI data were transformed to uncorrelated variables *via* principal component analysis. The variability across the study areas is summarized in principal components 1, 2, and 3, represented by the colors red, green, and blue, respectively.

The area selected for ENM calibration has a direct effect on the model results (24), resulting in models area-dependent. Thus, the calibration area must hypothesize the occurrence potential and the sampling effort of the organism in question (25). Based on Poo-Muñoz et al. (26), we used the average distance among available occurrences for *T. gondii* and *Bartonella* spp. to generate a buffer around occurrences in each region. The buffered area was used as model calibration region (26), assuming that this region provided a proxy of the landscape conditions contained across the sampled areas (8). Total areas considered for each selected regions are as follow: ~52,500 km^2^ for terrestrial area of California, ~105,400 km^2^ for Colorado divided in two polygons (Figure 1 left: ~64,700 km^2^ and right: ~40,700 km^2^), and ~43,000 km^2^ for terrestrial areas of Florida (Figure 1).

### Environmental Variables

Capturing fine-scale features of the landscape to understand the occurrence of pathogens is challenging and usually restricted to small study areas (27). A valuable alternative to landscape characterization is the use of satellite-derived remote sensing imagery. All objects emit radiation, at different intensities and wavelengths (28). This radiation can be characterized using satellite imagery from, for example, the MODerate-resolution Imaging Spectroradiometer sensor in the Terra satellite (29). These images offer low cost broad spatial coverage environmental information in the form of vegetation indexes (27), such as the Normalized Difference Vegetation Index (NDVI). The NDVI has proven representative of photosynthetic activity, biomass, net primary production, soil features, precipitations and humidity, and terrestrial landscapes in general. Thus, NDVI values have been associated with the distributional ecology and population dynamics of plants, invertebrates, birds, amphibians, ungulates, primates, carnivores, rodents, and reptiles in natural ecosystems; NDVI also provides information on changes in land use and soil humidity (27, 30).

Normalized Difference Vegetation Index data collected at 250 m spatial resolution at 16-day composites during 2005 in raster format were available from the Global Land Cover Facility (29). The resulting 21 original NDVI layers were reduced in number and collinearity *via* a principal component analysis (PCA) using ArcGIS 10.3 (31). We obtained new uncorrelated principal components (PC) with their respective descriptive values (e.g., correlation coefficients, eigenvalues, and eigenvectors). For the niche modeling procedure, we selected the PC summarizing at least 90% of the overall variance to capture a considerable amount of information from the original NDVI variables. The first three components were then utilized as axes to generate a three-dimensional environmental space as a proxy of Hutchinson’s duality to extract the environmental information of the geography (32) and were used to display occurrences in environmental terms. This environmental space was developed using NicheA 3.0 software (33), available at http://nichea.sourceforge.net/.

### Ecological Niche Modeling

We used Maxent 3.3.3k to generate the ecological niche models. Maxent is a machine learning tool developed to forecast species distributions with incomplete data (34). Maxent estimates the most uniform distribution of species occurrences compared with the available environmental background in the study area given constraints derived from the environmental data (35). Maxent also uses a regularization coefficient to increase or reduce the fit of the models to the available data, with a default value of 1 (36). We tested 20 regularization coefficients to find the best fit for our model. We used Akaike information criterion values corrected by sample sizes (AICc) to discriminate among models (37). This evaluation was developed using ENMTools 1.4.4 software (38). Specific settings in the final Maxent model included 100 bootstrap replicates with random seed and logistic output. The average of replicates in continuous format was converted to a binary format using a threshold value of *E* = 5%; this threshold aims to remove 5% of the calibration occurrences with the lowest logistic value (8).

Occurrence data were split into the three buffered study regions (i.e., California, Colorado, and Florida). Models were calibrated with all the occurrences in two regions, models were then transferred (neither clamping nor extrapolation allowed in Maxent) to the remaining region (39), and were then evaluated with the occurrences from such region (40). For example, we calibrated models using occurrence data for *T. gondii* from two regions (e.g., California and Florida) and evaluated predictions with occurrence data in the third region (e.g., Colorado). For *Bartonella* spp., due to the lack of occurrence records in Florida, we used one site (i.e., Colorado) to predict the other (i.e., California) and *vice versa*. This split configuration assured a fair evaluation of the models by using data independent from that used during model calibration. Maxent predictions were tested between the three study regions using partial receiver operating characteristic (Partial ROC) (41), a metric developed for ecological niche models to assess the correct prediction of independent evaluation occurrences and the proportion of area predicted suitable, against a null model (42). Partial ROC analyses were conducted using the Partial ROC metric (41, 43); parameters included 5% of omission, α < 0.05, 50% of random occurrences used for model testing, and 100 bootstrap iterations (41). Partial ROC estimates area under the curve (AUC) ratio values ranging between 0 and 2, with values above 1 (null model) resembling predictions better than by random expectations that are considered statistically significant (42).

## Results

**Figure 2:**
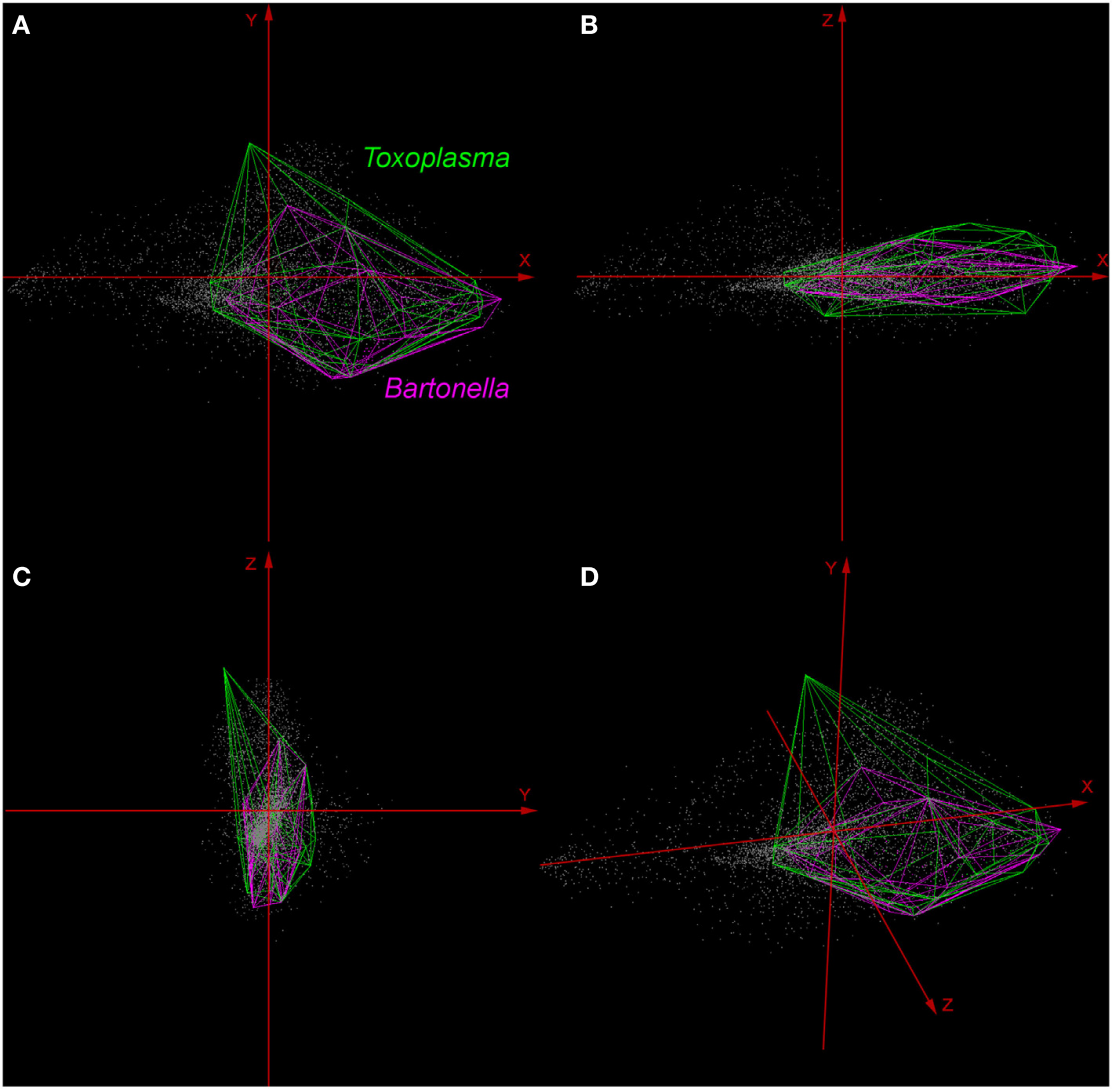
Distribution of *Toxoplasma gondii* and *Bartonella* spp. in a three-dimensional representation of the environmental space. All the available occurrences of *T. gondii* (green polyhedron) and *Bartonella* spp. (pink polyhedron) were displayed based on environmental values (gray points) available in California, Colorado, and Florida. Axes (red arrows) were constructed using principal components (PC) 1 (*X* axis), PC 2 (*Y* axis), and PC 3 (*Z* axis), which are the same variables represented in the right side of Figure 1. **(A)** View of the occupied niches based on PC 1 and 2. **(B)** View of niches based on PC 1 and 3. **(C)** View of niches using PC 2 and 3. **(D)** Three-dimensional view of species distributions based on PC 1, 2, and 3.

Environmental variables showed heterogeneous landscapes in spatial terms and collinearity among NDVI variables in temporal terms (Figure 1; Table S1 in Supplementary Material). For example, NDVI values in the summer (e.g., Julian days 193 and 209 in July in Table S1) showed low correlation with greenness with data from winter (e.g., days 1 and 17 in January in Table S1 in Supplementary Material). However, consecutive 16-day NDVI comparisons showed high correlation (e.g., Julian days 1 and 17, 17 and 33, and so on), with correlation coefficients ranging between 0.74 and 0.83 for comparisons between consecutive 21 layers (Table S1 in Supplementary Material). The first ten PC accumulated 90.77% of the overall information contained in the original 21 NDVI variables and were used for modeling (Table S2 in Supplementary Material). The first three components showed high environmental variability inside and between study areas, contained most of the information (80.37%) from the NDVI variables (Tables S2 and S3 in Supplementary Material), and showed differences in vegetation cover composition in California, Colorado, and Florida (Figure 1, right). Further, these three components were used to display the distribution of species in a three-dimensional virtual representation of the environmental space (Figure 2); here, the environmental distribution of both *T. gondii* and *Bartonella* spp. showed high overlap, despite the broader use of environmental conditions by *T. gondii* (Figure 2).

In all, 328 samples were positive for *T. gondii*, including 129 bobcats and 199 pumas across California, Colorado, and Florida (Figure 1). Two hundred thirty-four samples were positive for *Bartonella* spp. in 196 bobcats and 38 pumas from California and Colorado (Figure 1; Table 1). Models were calibrated using 291 single occurrence records for *T. gondii* and 189 occurrences for *Bartonella* spp. Models for both species required regularization coefficients other than the default value of 1 to have the best fit and lowest AICc: *T. gondii* required a regularization coefficient of 1.2, while the *Bartonella* spp. model required a regularization coefficient of 1.3 (Table S4 in Supplementary Material). Once calibrated, model evaluations showed that predictions between states were significantly better than a random model (AUC ratios above 1, *p* < 0.05) when data of *T. gondii* from California and Colorado were used to predict the location of this parasite in Florida (mean AUC ratio = 1.056, SD = 0.044), from Colorado and Florida to California (mean AUC ratio = 1.089, SD = 0.066), and when data from California and Florida were used to predict occurrences in Colorado (mean AUC ratio = 1.247, SD = 0.085) (Figure S1 in Supplementary Material). *Bartonella* spp. models calibrated in California were significantly predictive of the occurrence of this parasite in Colorado with AUC ratios above 1; similarly, models calibrated in Colorado significantly predicted *Bartonella* spp. in California (mean AUC ratio = 1.107, SD = 0.029) (Figure S1 in Supplementary Material).

**Table 1:** Positive cases by host (bobcat and puma), parasite (*Toxoplasma gondii* and *Bartonella* spp.), and region.

Infection	Host	California	Colorado	Florida	Total
*T. gondii*	Bobcat	51	10	5	66
	Puma	69	82	16	167
	Total	120	92	21	233
*Bartonella* spp.	Bobcat	102	31	N/A	133
	Puma	5	1	N/A	6
	Total	107	32	N/A	139
Co-infections	Bobcat	46	17	N/A	63
	Puma	22	10	N/A	32
	Total	68	27	N/A	95

*Samples from Florida were not tested for Bartonella spp*.

**Figure 3:**
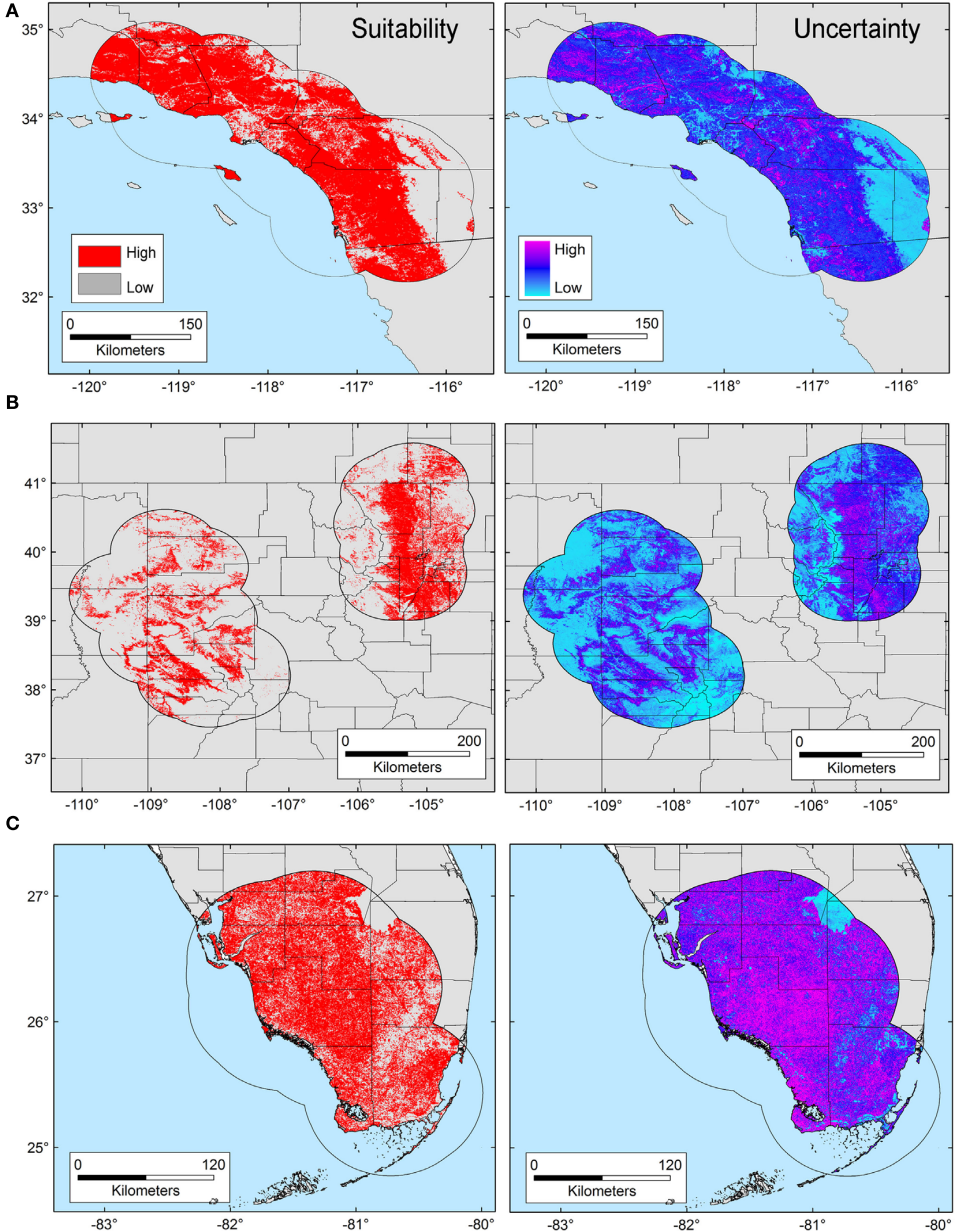
Ecological niche model of *Toxoplasma gondii*. Binary maps of *T. gondii* suitability (red) were developed for areas in California **(A)**, Colorado **(B)**, and Florida [**(C)**; left panel]. Uncertainty estimations based on the suitability differences among models (right panel) show areas of low (cyan) and high (pink) uncertainty as follows: California **(A)** from 3.31 × 10^−5^ to 0.42, Colorado **(B)** from 9.96 × 10^−6^ to 0.32, and Florida **(C)** from 8.13 × 10^−6^ to 0.3.

**Figure 4:**
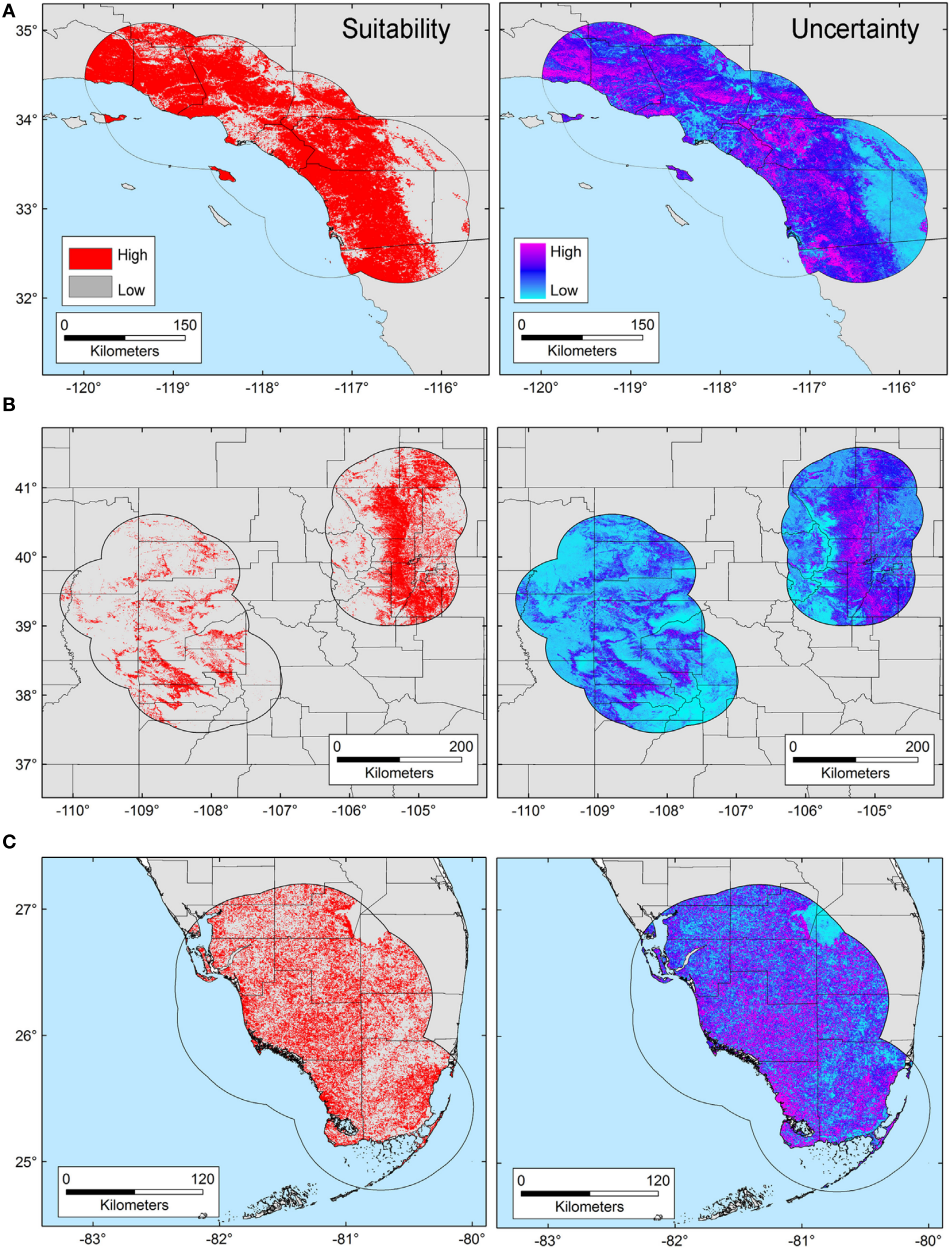
Ecological niche model of *Bartonella* spp. Binary maps of *Bartonella* spp. suitability (red) were developed for areas in California **(A)**, Colorado **(B)**, and Florida [**(C)**; left panel]. Uncertainty estimations based on the suitability differences among models (right panel) show areas of low (cyan) and high (pink) uncertainty as follows: California **(A)** from 5.77 × 10^−6^ to 0.37, Colorado **(B)** from 2.75 × 10^−6^ to 0.39, and Florida **(C)** from 8.59 × 10^−6^ to 0.39.

The *T. gondii* model identified suitable areas for this parasite, but also showed heterogeneity in uncertainty estimations across areas (i.e., predictions ranged from low uncertainty to high uncertainty in each area), with SD ranging between 8.13 × 10^−6^ (lowest) to 0.42 (highest; Figure 3). Binary models for *T. gondii* showed high proportion of suitability mainly in California (43.7% of the area) as compared with Colorado (35.8%) and Florida (20.5%); these predictions came with some variation in certainty (Figure 3A). Models also predicted isolated and limited suitability for both regions in Colorado, also with some variation evident in uncertainty, although these models were more confident in the places where *T. gondii* is unlikely to occur (Figure 3B). Florida showed wide suitability for this parasite across all the study areas (but with high uncertainty in suitability), except for consistent unsuitable predictions in Lake Okeechobee region (Figure 3C). Models for *Bartonella* spp. had a similar variation in predictions of suitable areas of pathogen occurrence, and uncertainty in predictions (SD ranging from 2.77 × 10^−6^ to 0.39; Figure 4). Our *Bartonella* spp. models predicted extensive suitability throughout California, with high certainty in unsuitable areas for *Bartonella* spp. occurrence (Figure 4A). It is notable that the area of uncertainty for *Bartonella* spp. in California was greater than for *T. gondii* (see Figures 3A vs. 4A). In Colorado, models predicted low *Bartonella* spp. suitability in the study area to the west with high certainty, but higher suitability to the east (Figure 4B). Even when no pathogen records were available to us for *Bartonella* spp. in Florida, our model predicted suitable conditions in specific sites across this region (Figure 4C) and with less uncertainty than *T. gondii*. In general terms, however, *Bartonella* spp. was predicted to be less widespread, as compared with *T. gondii* in Colorado and Florida.

## Discussion

Here, we illustrate the utility of a cutting-edge analytical tool that can be used to advance the understanding of the epidemiology of pathogens with complex lifecycles. Our modeling framework attempted to reconstruct the occupied niche of the parasites in question [*sensu* (8)]—the subset of the environmental space occupied by the species in the area studied. That is to say, the host species included in the study have broad home ranges [puma ~48.6 km^2^, bobcat ~30.7 km^2^ (44)] and occur through the Americas from Canada to Patagonia (puma) or across North America (bobcats), a typical characteristic of Felidae (45, 46). Thus, our representation of patterns of suitable areas for parasites is a high-resolution site and time specific reconstruction of risk. We found that although exposure to both *T. gondii* and *Bartonella* spp. was generally widespread in the study areas (Figure 1), *T. gondii* showed a broader distribution across environmental conditions than did *Bartonella* spp. (Figure 2), suggesting a broader niche for *T. gondii*. Although *Bartonella* spp. was not tested in the Florida samples, our niche model experiments suggest suitability in diverse areas of this state. We found that our models were most accurate in predicting areas where these parasites were least likely to occur. Specifically, the uncertainty, expressed as variability found in our Maxent predictions was the smallest for areas predicted unsuitable for *T. gondii* and *Bartonella* spp. (Figures 3 and 4).

Ecological niche models, and NDVI satellite imagery, proved to be useful to characterize the potential distribution of the selected pathogens at the landscape level, generating distribution maps for *T. gondii* and *Bartonella* spp. from exposure in wild felids. NDVI captures with high accuracy information of soil features, temperature conditions, and changes of humidity and precipitations as expressed in the structure of local vegetation (27); thus, allowing to capture the environmental signature of *Bartonella*-positive reservoirs associated with increments on precipitation, as is the case for some *Bartonella* species (47, 48). Environmental variables showed collinearity, and thus, using PC instead of the original NDVI variables mitigated Maxent overfit by reducing correlation and number of parameters employed by the model. The PCA allowed us to capture landscape variation, which was evident when the first three PC were displayed for each study area (Figure 1, right), suggesting that NDVI is a powerful tool for epidemiological studies aiming to forecast disease transmission risk at a habitat level (i.e., 250 m spatial resolution).

We had predictive success when applying ENM from one region to other, even though there were marked environmental differences among regions (Figure 1). Nevertheless, although all predictions among regions were significant, not all of our sites were equal in predictive abilities (Figure S1 in Supplementary Material). This highlights the key role that environment similarity can play between calibration and projection areas in Maxent. Potentially this supports the idea that Maxent predictions are more consistently suited for transference to similar environmental conditions (39). Further, we also show that NDVI environmental data are robust for reconstructing the environmental conditions suitable for pathogens, similar to more routine approaches using climate variables.

ENM applied to environmental dependent pathogens facilitates the identification of habitats of risk where collection of information has been lacking maybe due to limited sampling effort or other factors related to the detection of pathogens. It implies an advancement in understanding the distribution of pathogens beyond the use of data of their vectors or reservoirs. For example, *T. gondii* oocysts can be viable in the environment for up to 18 months (49), or potentially more importantly for these large felids, in their prey (rodents, lagomorphs, and cervids). *Bartonella* spp. easily survive in fleas whose abundance is associated with increasing humidity (50), and with microclimate conditions indirectly represented by NDVI, which could determine the distribution of *Bartonella* spp. between wildlife and domestic reservoirs (10). The ENM framework used here, including freely available vegetation data, with the presence-background Maxent algorithm, has the potential to be used to explore other environmental dependent pathogens. Our suitability maps of *T. gondii* and *Bartonella* spp. suggest that risk may exist in broad areas in the three states studied. Potential transmission may occur in the areas predicted suitable if hosts, the pathogen, and the vectors converge (Figures 3 and 4).

Despite the evident benefits, our approach is a simplification of two complex parasite systems. We based our interpretation of the pathogens’ niche from infected wild felids (i.e., bobcats and puma) in their sylvatic habitats, but may be missing other pieces of the epidemiological triangle. For instance, the distribution of intermediate hosts for *T. gondii* (such as rodents, lagomorphs, and cervids) (13) was not included in our models, nor was the presence of domestic cats (another definitive host) owing to insufficient data across all study areas. For *Bartonella* spp., we did not account for presence of vectors (e.g., fleas) (10), and thus, even when we anticipate suitable conditions for the parasite occurrence, suitable conditions for vectors could limit the occurrence of *Bartonella* spp. in certain areas. Moreover, we modeled *Bartonella* spp. at genus level under the assumption of niche conservatism, which proposes that species phylogenetically close will share ecological niche characteristics, and that intraspecific differentiation of niches is challenging (51, 52). Although our diagnostics tests have proven effective for these wild felids, there could exist a small number of false-positive and false-negative results, we assumed that this proportion would not change the general patterns of the findings.

Ecological niche models of both parasite species based on hosts from wild areas revealed that our models were a proxy of the sylvatic cycle of both parasites; however, these pathogens might also occur in urban areas, which are not often frequented by puma or bobcats. *T. gondii* can also occur in urban environments given its adaptability and host generalization as a result of its broad ecological niche (53). Nonetheless, Lélu et al. (54) suggest *T. gondii* is likely to be less prevalent in urban areas owing to reduced transmission through the food chain, a conclusion supported by our work in these study areas where domestic cats are restricted to urban areas, wild felids avoid urban areas, and *T. gondii* has a higher prevalence in wild felids (12). Conversely, our previous research shows a strong positive relationship between urbanization and exposure to *Bartonella* spp. (12), suggesting that these bacteria persist in stable homogeneous urban landscapes. Future research should include the urban component in the distribution of *T. gondii* and *Bartonella* spp. parasites for a broader characterization of the ecological potential of both parasites in natural and impervious surfaces in North America.

Previous studies have demonstrated niches of pathogens independent of potential reservoir distributions (55), thus showing that the modeling of pathogens-only provides accurate forecasts of disease transmission risk. These cutting-edge available tools of disease modeling are worthy of exploration to generate further fine-scale hypotheses to advance our knowledge of the environmental component of infectious disease transmission chains (9). Although the occurrences of the two pathogens were explored in wildlife, they are also zoonotic, so the results of this study have implications for human, as well as domestic and other wild animals’ health. NDVI and longitudinal epidemiological studies can help address questions not only about the prevalence of *Bartonella* spp. and *T. gondii* in the environment, but also can allow us to identify suitable habitats for their presence, and in turn, forecast into the future as these methods can incorporate the effects of land use change to understand the ecology of infectious diseases, particularly environmentally dependent forms, before outbreaks occur.

## Author Contributions

LE and DR-A designed the experiments, developed the analyses, and co-wrote the manuscript. SC and MC designed the study and co-wrote the manuscript. SV, KC, and ML provided the data and co-wrote the manuscript.

## Conflict of Interest Statement

The authors declare that the research was conducted in the absence of any commercial or financial relationships that could be construed as a potential conflict of interest.
