# Supplementary material for: Inferring the Ecological Niche of Toxoplasma gondii and Bartonella spp. in Wild Felids
Source: Front Vet Sci. 2017 Oct 17;4:172. doi: 10.3389/fvets.2017.00172 (PMC5650989; doi:10.3389/fvets.2017.00172)
Supplement: Supplementary file 1 [file Data_Sheet_1.pdf]

frontiers

Supplementary Material

Inferring the ecological niche of *Toxoplasma gondii* and *Bartonella* spp. in wild felids

Luis E. Escobar[1,2,3*], Scott Carver[4†], Daniel Romero-Alvarez[5†], Sue VandeWoude[5], Kevin R. Crooks[6], Michael R. Lappin[7], Meggan E. Craft[1]

[1]Department of Veterinary Population Medicine, University of Minnesota, Minneapolis, Minnesota 55455, United States

[2]Department of Fisheries, Wildlife and Conservation Biology, University of Minnesota, St. Paul, MN, United States

[3]Department of Fish and Wildlife Conservation, Virginia Tech, Blacksburg, VA, United States

[4]School of Biological Sciences, University of Tasmania, Hobart, Tasmania 7001, Australia

[5]Department of Ecology and Evolutionary Biology, University of Kansas, Lawrence, KS, United States

[6]Department of Microbiology, Immunology and Pathology, Colorado State University, Fort Collins, Colorado 80523, United States

[7]Department of Fish, Wildlife, and Conservation Biology, Colorado State University, Fort Collins, Colorado 80523, United States

[8]Department of Clinical Sciences, Colorado State University, Fort Collins, Colorado 80523, United States

[†] Contributed equally.

*** Correspondence:**
Luis E. Escobar, lescobar@umn.edu, escobar1@vt.edu

Supplementary Figures

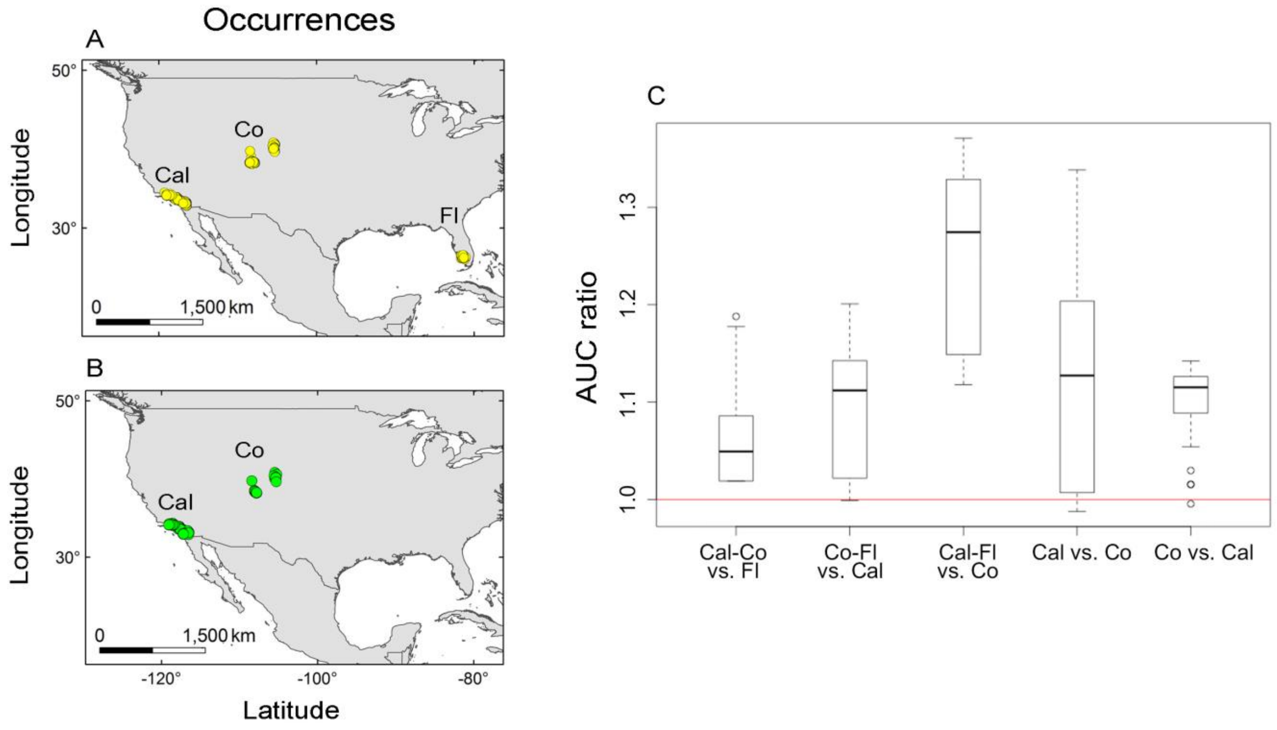

Supplementary Figure S1. Exploring *Toxoplasma gondii* and *Bartonella* spp. predictability between regions.

A. *T. gondii* occurrences (yellow points) in California (Cal), Colorado (Co), and Florida (Fl). **B.** *Bartonella* spp. occurrences (green points) in California (Ca), and Colorado (Co). **C.** AUC ratio from different Partial ROC evaluations represented as boxplots for their respective *T. gondii* and *Bartonella* spp. For *T. gondii* Cal-Co vs. Fl resulted in a mean AUC ratio = 1.056, standard deviation (sd) = 0.044; Co-Fl vs. Cal, mean AUC ratio = 1.089, sd = 0.066; and Cal-Fl vs. Co mean = 1.247, sd = 0.085. For *Bartonella* spp., Cal vs. Co mean AUC ratio = 1.134, sd = 0.095; and Co vs. Cal, mean AUC ratio = 1.107, sd = 0.029. Note that all comparisons reported most AUC ratios above 1—the null model (red line, Peterson, 2012; Peterson et al., 2008).

Supplementary Tables

Supplementary Table S1. Correlation matrix of original environmental variables.

NDVI layers are categorized by the first day of 16-day data collection in Julian days. Layers were identified as those with high (red), medium (yellow), and low (green) correlation coefficient values.

Days	1	17	33	49	65	81	97	113	129	145	161	177	193	209	225	241	257	273	289	305	321
**1**	1	0.8	0.75	0.74	0.7	0.72	0.65	0.64	0.55	0.44	0.38	0.36	0.36	0.33	0.35	0.37	0.44	0.43	0.56	0.58	0.62
**17**		1	0.82	0.81	0.76	0.77	0.71	0.7	0.6	0.48	0.42	0.42	0.4	0.36	0.38	0.4	0.49	0.48	0.61	0.65	0.69
**33**			1	0.8	0.77	0.8	0.74	0.71	0.59	0.49	0.42	0.39	0.38	0.34	0.38	0.42	0.46	0.45	0.59	0.62	0.67
**49**				1	0.77	0.79	0.72	0.7	0.56	0.45	0.39	0.36	0.34	0.31	0.34	0.37	0.42	0.42	0.55	0.59	0.63
**65**					1	0.79	0.74	0.69	0.6	0.45	0.35	0.36	0.31	0.3	0.35	0.37	0.39	0.4	0.49	0.55	0.59
**81**						1	0.83	0.79	0.64	0.54	0.44	0.38	0.36	0.34	0.39	0.45	0.44	0.44	0.57	0.6	0.67
**97**							1	0.8	0.7	0.6	0.51	0.46	0.43	0.39	0.45	0.51	0.5	0.49	0.59	0.63	0.67
**113**								1	0.76	0.68	0.6	0.53	0.51	0.48	0.51	0.55	0.55	0.55	0.67	0.69	0.71
**129**									1	0.76	0.71	0.69	0.64	0.6	0.63	0.63	0.66	0.62	0.7	0.71	0.68
**145**										1	0.79	0.74	0.72	0.69	0.7	0.74	0.7	0.65	0.73	0.7	0.65
**161**											1	0.79	0.77	0.73	0.71	0.74	0.74	0.67	0.75	0.7	0.64
**177**												1	0.8	0.78	0.75	0.77	0.79	0.71	0.74	0.69	0.6
**193**													1	0.77	0.74	0.78	0.78	0.7	0.74	0.68	0.6
**209**														1	0.74	0.76	0.75	0.68	0.7	0.64	0.56
**225**															1	0.77	0.74	0.66	0.69	0.64	0.57
**241**																1	0.79	0.7	0.75	0.68	0.63
**257**																	1	0.74	0.78	0.73	0.67
**273**																		1	0.74	0.7	0.65
**289**																			1	0.83	0.8
**305**																				1	0.8
**321**																					1

Supplementary Table S2. Eigenvalues of the principal component analysis from the NDVI data.

The first ten principal components (bold) contained >90% of the overall variance and were used to construct the multivariate environmental space to develop the ecological niche models. The first three components were used to display the distribution of parasites in three-dimensional environmental space (see Fig. 2).

PC	Eigenvalue	Percent of Eigenvalues	Eigenvalues accumulation
**1**	1031.78	62.41	62.41
**2**	248.74	15.05	77.45
**3**	48.25	2.92	80.37
**4**	37.2	2.25	82.62
**5**	28.58	1.73	84.35
**6**	24.18	1.46	85.81
**7**	22.61	1.37	87.18
**8**	21.01	1.27	88.45
**9**	19.55	1.18	89.63
**10**	18.7	1.13	90.77
11	16.58	1	91.77
12	16.52	1	92.77
13	16.37	0.99	93.76
14	15.38	0.93	94.69
15	14.58	0.88	95.57
16	14.08	0.85	96.42
17	13.47	0.81	97.24
18	12.78	0.77	98.01
19	12	0.73	98.74
20	10.95	0.66	99.4
21	9.96	0.6	100

Supplementary Table S3. The eigenvector coefficients of a standardized principal component analysis of the original 21 NDVI variables.

NDVI layers are categorized by the first day of the 16-day data collection in Julian days.

Variable	PC 1	PC 2	PC 3
Day 001	0.21135	-0.29277	-0.33271
Day 017	0.23326	-0.30283	-0.27235
Day 033	0.2155	-0.28883	-0.06093
Day 049	0.20557	-0.30667	-0.05727
Day 065	0.19423	-0.29812	0.1721
Day 081	0.20529	-0.2711	0.2414
Day 097	0.20086	-0.18948	0.35873
Day 113	0.20351	-0.12498	0.30847
Day 129	0.20501	0.00985	0.31139
Day 145	0.21775	0.13072	0.34872
Day 161	0.21947	0.20648	0.17144
Day 177	0.21718	0.24001	0.02109
Day 193	0.21717	0.25547	-0.05953
Day 209	0.20897	0.26642	-0.05677
Day 225	0.21138	0.23346	0.08988
Day 241	0.22388	0.2254	0.07488
Day 257	0.22959	0.196	-0.19638
Day 273	0.20639	0.14666	-0.20529
Day 289	0.256	0.09477	-0.24302
Day 305	0.24454	0.03286	-0.20772
Day 321	0.24398	-0.04755	-0.21126

Supplementary Table S4. Akaike information criterion corrected by sample size (AICc).

AICc values for different regularization coefficients for model fit in Maxent. Occurrences for *Toxoplasma* = 291, *Bartonella* = 189. Number of variables used for parametrization = 10. In bold are the lowest AICc value representing the regularization coefficient with the best fit.

	Toxoplasma gondii			***Bartonella spp.***		
**Regularization Coefficients**	**Log likelihood**	**Parameters**	**AICc**	**Log likelihood**	**Parameters**	**AICc**
0.1	-4267.64	247	10560.69	-2886.52	226	20882.76
0.2	-4285.52	231	10149.55	-2907.28	207	9540.56
0.3	-4308.62	200	9650.32	-2928.08	182	7526.28
0.4	-4332.27	174	9410.57	-2953.25	154	6818.81
0.5	-4351.23	158	9315.76	-2972.10	127	6504.92
0.6	-4372.44	144	9261.07	-2989.87	105	6363.66
0.7	-4387.89	131	9214.22	-3004.95	96	6337.84
0.8	-4403.45	115	9162.75	-3019.47	82	6293.09
0.9	-4414.62	99	9114.08	-3034.11	76	6294.77
1	-4426.67	93	9114.05	-3044.19	70	6289.35
1.1	-4434.48	73	9057.49	-3052.69	62	6275.06
1.2	-4440.37	68	**9052.98**	-3057.69	54	6256.56
1.3	-4446.88	66	9059.64	-3058.84	52	**6252.14**
1.4	-4452.02	66	9069.92	-3062.84	50	6253.54
1.5	-4455.69	68	9083.61	-3067.50	48	6256.43
1.6	-4460.56	64	9080.76	-3071.71	44	6252.36
1.7	-4464.15	63	9084.84	-3076.16	46	6267.45
1.8	-4468.88	58	9079.20	-3080.09	43	6266.10
1.9	-4473.88	59	9092.18	-3079.87	40	6256.73
2	-4483.20	56	9101.95	-3081.18	41	6262.30
